# Button Battery-Induced Necrotizing Keratoconjunctivitis: Case Report

**DOI:** 10.1155/2022/7878031

**Published:** 2022-12-10

**Authors:** Nima Koosha, Leila Babaei, Mohsen Pourazizi

**Affiliations:** Isfahan Eye Research Center, Department of Ophthalmology, Isfahan University of Medical Sciences, Isfahan, Iran

## Abstract

We report a case of a 2-year-old girl who presented to the ocular emergency department with a button battery retained in the inferior fornix of the left eye for more than 48 hours. The child developed necrotizing keratoconjunctivitis, which was treated with antibiotics, amniotic membrane graft, prompt removal of button battery, and other supportive measures.

## 1. Introduction

The usage of button batteries (BBs) has become more common in recent years to power electronic equipment and toys. On one hand, BBs are increasingly used in devices including electronic games, watches, and new electronic toys. On the other hand, the advent in size reduction and also the smooth and shiny appearance of BB make them quite attractive and interesting to young children, and the number of serious injuries or deaths as a result of small BBs has increased in the last decade [1, 2].

BBs can be extremely dangerous for children due to electrochemical mechanism of injury [1, 3, 4]. If BBs are swallowed or placed in the any orifice of the body, they can cause significant damage to the mucosa and potentially cause long-term complications [1–3]. The clinical course of a child with a BB depends on several factors including the location, duration of exposure, battery size, power of BB, remaining voltage in the battery, and chemical composition of the battery [1, 2].

Locations for BBs injury in children include aerodigestive tract, nose, vagina, and ear [2, 5]. Embedded BB in the eye is very rare, and there is limited report of ocular surface damage from BBs [6–9]. The purposes of this report are to present our experience in the diagnosis and management of a new case of BB foreign body in a young child and review the related literature.

## 2. Case Presentation

A 2-year-old girl was brought to emergency department of the referral eye center of Feiz Hospital affiliated to Isfahan University of Medical Sciences, Isfahan, Iran, by her mother with complaints of redness, tearing, and pain. The patient also had bloody stained discharge in her left eye. The interval between initiation of ocular symptoms and first visit was about 48 hours. The patient was otherwise healthy with no significant medical or ophthalmic history. Prior to injury, she had been left playing alone. There was no history of ocular trauma.

**Figure 1:**
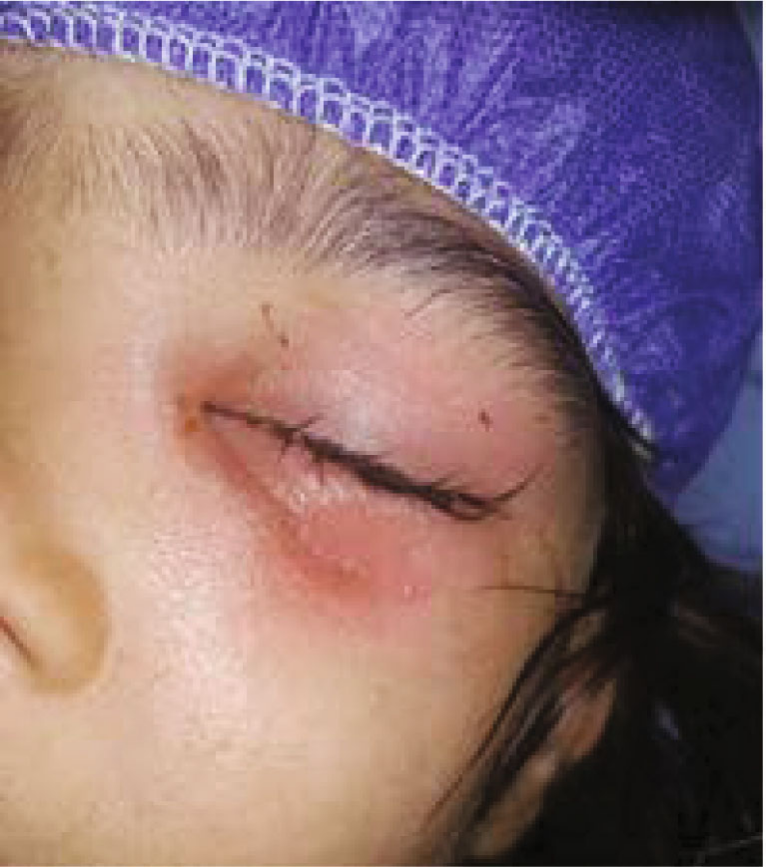
Edema of left upper and lower eyelids.

In the emergency room, physical exam revealed stable vital signs and swollen left upper and lower eyelids (Figure 1).

**Figure 2:**
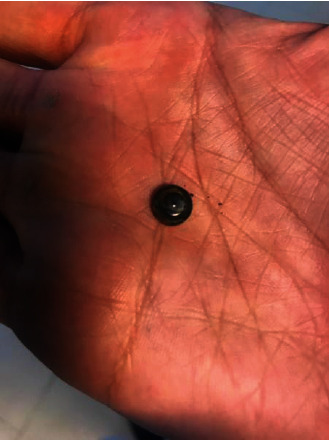
Removed alkaline button battery from left inferior fornix in a child.

Slit-lamp examination in the emergency room showed a disc foreign body in the inferior fornix. The pupil was reactive with normal size. The extraocular movements were normal. The foreign body was an alkaline 1.5 volt, 6 millimeter BB obtained from a musical toy. The button battery was removed in the emergency room, and a chemical ocular injury in the left eye was suspected (Figure 2).

**Figure 3:**
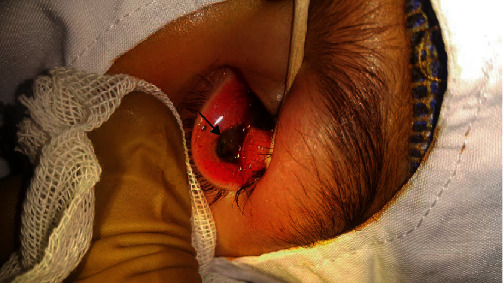
Necrosis in the inferior fornix of the left eye due to button battery exposure (black arrow).

Due to poor cooperation of child in the emergency room, the pH of the ocular surface tear film in the affected eye was not evaluated. The eyes were irrigated with balanced salt solution. Fluorescein solution was applied over the ocular surface to identify the extent of injury. Multiple scattered epithelial defects (ED) and an area of epithelial necrosis in inferior fornix were discovered. Apart from the corneal ED, the inferior limbus from 4 to 8 o'clock was ischemic and conjunctiva also had evidence of chemical toxicity and necrosis inferiorly (Figure 3).

The anterior chamber was quiet, and the sclera was intact. The rest of the anterior segment examination was unremarkable in the left eye, and the posterior segment was within normal limits. To rule out the rare possibility of any intraorbital foreign body, an axial and coronal CT were also done, which revealed air in the inferior orbital space-like necrotic tissue. A complete ophthalmic exam was performed under general anesthesia in the operating room. Intraocular pressure was in a normal range.

During exploration of the conjunctiva, severe tissue destruction was noted in the lower palpebral conjunctiva caused by the alkaline BB. In the operation room, irrigation was continued until normal surface pH unit.

All necrotic tissue of cornea and conjunctiva was debrided. A commercial amniotic membrane was applied to cover the raw surface as a biological bandage and to promote epithelialization. The amniotic membrane is secured with multiple 8-O Vicryl™ absorbable sutures. The patient was given systemic intravenous antimicrobial treatment (cefazolin; 1 mg/kg body weight) postoperatively for 72 hours.

**Figure 4:**
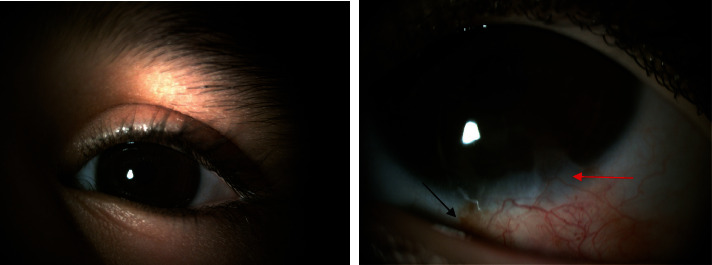
After 6-month follow-up. Sectoral superficial pannus formation in the inferior temporal quadrant with distinct borders, pannus formation (red arrow), and an adhesion band formation (black arrow).

The eye treated with topical ciprofloxacin every 4 hours, topical betamethasone every 6 hours, and a preservative-free artificial tear frequently. The patient's condition continuously improved, and the epithelial defect had completely healed. Both the cornea and conjunctiva gradually became normal without fluorescein staining after 2 weeks. All medications tapered off within 4 weeks. On 6-month follow-up, there was no sign of limbal deficiency, and the clarity of the cornea was preserved (Figure 4).

## 3. Discussion

We report a case of ocular BB foreign body leading to necrotizing keratoconjunctivitis. The importance of this report lay on (1) the organ-threatening consequence of a delay in removing the BB and (2) the importance of applying AMT when there is a significant tissue loss for repair. Evidence of BB exposure was apparent by the ocular surface necrotizing injury, with confirmation upon discovery of a small BB in the inferior fornix of the left eye.

A toxic keratoconjunctivitis has been reported to be caused by a wide range of toxic foreign body including chemical powder, caustic agents, remnants of shampoo and cosmetic lotions, and chlorinated pool water. Although foreign bodies are usually removed from the ocular surface by the protective mechanisms, embedded foreign bodies can potentially be serious toxic ocular foreign bodies and cause tissue destruction [10].

If the battery is damaged or shows any leakage, the possible mechanism is chemical injury; and if the battery is intact with considerable charge for example in new toys, the possible mechanisms like electrical burns or generation of hydroxide can be considered [1, 3–5].

In our case, the suspected mechanism of injury was likely chemical as the removed BB was not intact.

Exudation of tissue fluids caused by a burn injury creates a moist environment. In vitro studies have shown that spontaneous leakage of electrolyte solution occurs when alkaline batteries are exposed to moisture. The leaked alkaline electrolyte solution can penetrate deeply into tissues producing a liquefying necrosis. This results in dissolution of protein and collagen, saponification of lipids, dehydration of tissue cells, and consequential extensive tissue damage [1, 4].

BBs ocular injury is a rarely reported condition in the literature. Some reports revealed ocular toxicity or injury after exposure to a BB [6–9] (Table 1). Given the unusual site of BBs as a foreign body in eye, occurrences of ocular toxicity due to BBs are rare; therefore, some ophthalmologists may never have encountered ocular BBs injury and would not recognize the clinical and ocular characteristics of retained BBs in the eye.

In 2011, in an experimental study, a button battery was placed on an eyeball of a pig. The result of the study demonstrated corneal opacity development in five minutes [6]. Ogasawara et al. report a case of button battery-induced ocular injury in a 3-year-old girl that presented with corneal opacity and conjunctival injection. Over several months, scar tissue was replaced by secondary pterygium [6]. In a similar report, Mazer-Amirshahi et al. report an 18-year-old girl with severe conjunctival ulceration, subconjunctival hemorrhage, vitreous opacification, and a partially dilated pupil associated BB injury. The patient was treated with topical antibiotics, steroids, and a daily rodding procedure using glass rod to separate the bulbar and tarsal conjunctiva to prevent the tarsal conjunctival scar and finally healed over the subsequent 6 months [7]. In another case, Ratnarajan et al. report severe ocular injury from a BB with a delayed presentation after about 18 hours exposure to the BB [8]. Khan et al. report a rapid onset case of severe ocular injury after exposure to a BB. Their case presented with swelling and redness of the eye within 3 hours after exposure. Due to severe ocular injury, symblepharon formation occurred [9].

Since only few hours may be needed to result in major complications, the most important issue in management of BB foreign bodies is rapid diagnosis and prompt removal of any BB contacting any mucosal membranes [1, 4]. In our patient, the BB remain for more than 48 hours and induced severe necrotizing injury. The toxic reaction in our patient was more severe possibly due to the long-lasting retained BB within the lower eyelid and continuous release of toxic electrolytes in an alkaline condition, which could contribute to additional ocular surface damage.

A previous study reported successful treatment of BB ocular burn using steroid. Steroid plays a major role in controlling inflammation, especially in the acute stage and subsequently prevent of development of symblepharon and deformation of the ocular surface. We also used amniotic membrane to promote the healing process that had a favorable result in our patient.

Transplantation of amniotic membrane has been shown to promote epithelialization and reduce inflammation, scarring, and neovascularization [11]. Our report is also not without limitations. The study was a single case report; and therefore, generalisation of this report is cautioned by the authors. Another limitation of current report included absences of more follow-up period.

## 4. Conclusion

Our report provides new insight in clinical presentation and the significance of early removal, copious irrigation, medical treatment, and the unique treatment of amniotic membrane as an adjunct to the BB injuries in the eye. Due to the common use of these batteries, physicians should be more aware of the serious risks associated with BBs. Since there is no standard of protocol for treatment of BB-induced ocular injury, it seems prevention is the best approach for to be safe from BB-induced ocular injury.

## Figures and Tables

**Table 1:** Ocular injury after exposure to a BB in the literature.

Case no.	Author(s)/years	Patient age/sex	Latency until examination	Ocular manifestations	Management	Prognosis
1	Ogasawara et al./2011 [6]	3/F	8 hours	(i) Hard and polished white opacity on the cornea (ii) Conjunctival injection	(1) Removal button battery (2) Ocular surface irrigation	Improvement of the opacity gradually secondary pterygium formation
2	Mazer-Amirshahi et at./2013 [7]	18/F	90 min	(i) Conjunctival ulceration (ii) Subconjunctival hemorrhage (iii) Vitreous opacification (iv) Partially dilated pupil	(1) Removal button battery in the operating room (2) Ocular surface irrigation (3) Topical antibiotics (4) Topical steroids (5) Daily rodding procedure using glass rod to separate the bulbar and tarsal conjunctiva	Healing over the subsequent 6 months, normal visual acuity
3	Ratnarajan et al./2013 [8]	2/F	18 hours	(i) Lower eyelid edema and erythema (ii) Severe eye pain	(1) Removal button battery (2) Daily rodding procedure for 3 days (3) Betamethasone 1% ointment four times a day (gradually tapered over 3 months)	Symblepharon between the bulbar and tarsal conjunctiva without eye movements restriction
4	Khan et al./2014 [9]	2/F	3 hours	(i) Eye lid erythema and swelling with a pseudoptosis	(1) Removal button battery	Symblepharon formation
5	Our case/2021	2/F	48 hours	(i) Upper and lower eyelid edema and erythema (ii) Tearing (iii) Severe pain (iv) Bloody discharge	(1) Removal button battery (2) Ocular surface irrigation (3) Topical betamethasone (4) Topical ciprofloxacin (5) Systemic antibiotic	Improvement with no sign of limbal deficiency on 6-month follow up

## Data Availability

Data are available on request.
